# Soft Tissue Sarcoma with Lower Limb Impairment: Development of a Specific Rehabilitation Protocol Based on Demolitive and Reconstructive Surgery Types

**DOI:** 10.3390/jcm13237023

**Published:** 2024-11-21

**Authors:** Caterina Galluccio, Marco Germanotta, Sergio Valeri, Beniamino Brunetti, Bruno Vincenzi, Stefania Tenna, Chiara Pagnoni, Rossana Alloni, Michela Angelucci, Rosa Salzillo, Marco Morelli Coppola, Alice Valeri, Roberto Passa, Francesca Falchini, Arianna Pavan, Laura Cortellini, Stefania Lattanzi, Irene Giovanna Aprile

**Affiliations:** 1 IRCCS Fondazione Don Carlo Gnocchi Onlus, 50143 Florence, Italy; cgalluccio@dongnocchi.it (C.G.); ffalchini@dongnocchi.it (F.F.); apavan@dongnocchi.it (A.P.); lcortellini@dongnocchi.it (L.C.); slattanzi@dongnocchi.it (S.L.); iaprile@dongnocchi.it (I.G.A.); 2 Operative Research Unit of Soft-Tissue Sarcomas Surgery Department, Fondazione Policlinico Universitario Campus Bio-Medico, 00128 Rome, Italy; s.valeri@policlinicocampus.it (S.V.); c.pagnoni@policlinicocampus.it (C.P.); r.alloni@policlinicocampus.it (R.A.); michela.angelucci@unicampus.it (M.A.); r.passa@unicampus.it (R.P.); 3 Operative Research Unit of Plastic-Reconstructive and Aesthetic Surgery, Fondazione Policlinico Universitario Campus Bio-Medico, 00128 Rome, Italy; b.brunetti@policlinicocampus.it (B.B.); s.tenna@policlinicocampus.it (S.T.); r.salzillo@policlinicocampus.it (R.S.); m.morellicoppola@policlinicocampus.it (M.M.C.); 4 Operative Research Unit of Medical Oncology, Fondazione Policlinico Universitario Campus Bio-Medico, 00128 Rome, Italy; b.vincenzi@policlinicocampus.it; 5 Operative Research Unit of Plastic-Reconstructive and Aesthetic Surgery, Università Campus Bio-Medico, 00128 Rome, Italy; alice.valeri@alcampus.it

**Keywords:** soft tissue sarcoma, rehabilitation, surgery, plastic surgery procedures, protocol

## Abstract

**Background/Objectives:** Soft tissue sarcomas (STSs) are extremely uncommon tumors with a high rate of local recurrence that often require very demolitive surgery. The aim of our study is to propose a specific rehabilitation protocol for patients with STSs, based on the kind of demolition and reconstructive surgery performed, and evaluate its effects. **Methods**: The protocol was developed on the basis of the clinical experiences of physiatrists and surgeons, as well as data from the literature, recommending timelines for postural steps, verticalization, walking, and therapeutic exercises, in accordance with wound healing times and in order to prevent complications from disuse and immobility. The modified Barthel Index Scale (mBI), the Numeric Rating Scale (NRS), the Adapted Patient Evaluation Conference System (APECS), and the 10 Meter Walk Test (10 MWT) were used to clinically evaluate patients before and after the rehabilitation treatment. **Results**: Thirty-one patients with primary STSs were enrolled. Following the rehabilitation program, we found a statistically significant improvement in mBI (*p* < 0.001), APECS (*p* ≤ 0.001), and NRS (*p* = 0.001). In a subgroup of patients (*n* = 18) assessed with the 10 MWT, a statistically significant increase in walking speed was observed (*p* = 0.012). **Conclusions**: Patients who completed rehabilitation following the proposed protocol, customized according to the surgical intervention type, demonstrated marked improvements in motor performance, ability in daily activities, walking, and pain. The proposed protocol can assist the multidisciplinary team of surgeons, oncologists, and rehabilitators in maintaining alignment on patient management, thereby ensuring clear indications regarding the activities that patients can and cannot undertake during the recovery period.

## 1. Introduction

Soft tissue sarcomas (STSs) are rare and heterogeneous tumors that account for approximately 1% of all malignant neoplasms in adults [1]. Their localization is variable, occurring in the trunk, limbs, or abdomen. The extremities are the most frequent site of involvement, accounting for approximately 45% of cases [2].

These tumors are classified into more than 80 distinct histotypes, exhibiting highly variable behaviors [3]. This heterogeneity requires a comprehensive biopsy and a multidisciplinary discussion involving general and plastic surgeons, anatomopathologists, clinical oncologists, and radiologists to determine the most appropriate treatment for each case [4]. It is important to note that STSs are a rare condition, with a correspondingly limited number of clinicians with expertise in their treatment. This has led to the establishment of specialized centers where experts in the field can cooperate in multidisciplinary teams [5].

The treatment of this type of tumor is also varied, depending on the aggressiveness, extent, histotype, and age of the patient. For localized tumors, the initial approach is often surgical, which may involve extensive surgical resection. In some cases, chemotherapy or radiation therapy may be employed in conjunction with surgery. The choice of treatment depends on the characteristics of the tumor [6]. The standard of care and goal to be achieved is limb salvage, but in a small percentage of cases, amputation may be necessary depending on the extent of the tumor and its features.

STSs, however, have a five-year survival rate of 55 to 65% with a high incidence of local recurrence [7]. For this reason, current guidelines recommend wide excision with an oncological margin based on histologic type [6]. The loss of a large amount of muscle tissue may necessitate reconstructive plastic surgery treatments.

Following surgical resection of a large STS, various reconstructive techniques may be utilized depending on the location and extent of the resected tissue, as well as the chemotherapy or radiotherapy administered or planned [8].

In the case of a superficial lesion, primary closure or soft tissue coverage can be performed with a simple skin graft. If primary closure is not feasible or if a deep lesion necessitates complete removal with exposing the bones, vascular structures, or nervous system, it is preferable to cover these structures. The selected method may encompass (a) local perforator flaps (LPF), for moderate-sized tissue excision in patients not undergoing radiation therapy; (b) free flap (FF), for extensive tissue removal or in patients undergoing radiation therapy; (c) free functional muscle transfer (FFMT), when an extensive amount of tissue is removed and restoration of tissue function is required [8].

Recent advances in the treatment of this disease have led to an increase in survival rates, but this has also resulted in an increase in mid- to long-term disability. Indeed, the consequences of the disease and its treatments impact motor function, activities of daily living, pain, and, therefore, quality of life [9].

Furthermore, the disease can result in several complications, such as lymphedema (secondary to surgery or radiotherapy), fatigue and sleep disorders, reduced aerobic exercise capacity, and mood disorders such as depression [10]. All or some of these possible consequences require a tailored rehabilitation program.

The role of rehabilitation in patients with cancer, regardless of the site, is widely recognized in the literature [11]. In fact, exercise plays a pivotal role in prevention, sequelae management, symptom control, and the return to daily life in all types of cancers [12].

Some studies suggest the importance of a rehabilitation pathway even prior to surgery in the cancer patient (pre-rehabilitation) [13,14]. In STSs, preoperative poor performance status has been demonstrated to be a factor associated with a worse prognosis [15].

However, the existing literature contains few studies focusing on the rehabilitation of patients with STSs. For instance, Cody C. Andrews and colleagues underlined the significance of inpatient rehabilitation for patients who have undergone amputation, particularly with regard to prosthesis selection and adaptation [16]. In contrast, for patients who have undergone limb preservation, rehabilitation should aim to restore range of motion and strengthen muscles around the resected tumor area [17]. Tobias and Gillis provided a comprehensive account of the rehabilitation plan of a patient, delineating the various stages, from the pre-rehabilitation phase to the sub-acute phase, and finally to the chronic phase, before returning home. They underscored the significance of an individualized rehabilitation pathway, emphasizing its foundation in the specific tissue removed during surgery, potential complications, and the administration of radio/chemotherapy [14].

Notably, patients with STSs often receive radiation therapy before or after surgery, which might prolong the healing process [18]. According to Peter Hohenberger and Matthias H.M. Schwarzbach, radiation therapy actually slows down the proliferation phase and reduces collagen formation, which is why actinic damage to the microvasculature is particularly evident after irradiation [19]. This may account for any delays and the different timing in achieving rehabilitation goals.

Currently, research on the efficacy of rehabilitation for patients suffering from sarcomas is limited and there is a lack of personalized rehabilitation protocols that consider the type of intervention which the patient has undergone.

The purpose of our study is, therefore, to suggest a specific rehabilitation protocol for patients with lower extremity STSs, based on demolition surgery and type of reconstructive surgery performed, and assess its outcomes.

## 2. Materials and Methods

### 2.1. Study Design

This interventional study analyzes patients with soft tissue sarcoma (STS) recruited for rehabilitation treatment at the Santa Maria della Provvidenza Center of the Fondazione Don Carlo Gnocchi Onlus in Rome.

Informed consent was obtained from all subjects involved in the study, after a detailed explanation of the study’s aims and rehabilitation protocols. The study was approved by the Ethics Committee of the Fondazione Policlinico Universitario Campus Bio-Medico (Protocol number PAR 77.22 OSS) and registered at ClinicalTrials.gov (ID NCT06282237).

### 2.2. Subjects

This study included thirty-one adult patients with soft tissue sarcoma of the lower extremities or retroperitoneum (with iliopsoas muscle involvement and possible femoral nerve injury) who were undergoing demolitive surgery with curative intent. Some patients subsequently underwent reconstructive surgery, including the use of pedicled flaps, free flaps, and functional flaps. Patients with recurrent tumors, metastatic disease, and those receiving palliative surgery and amputations were excluded from the study.

### 2.3. Rehabilitation Protocol

All patients participated in two 50-min rehabilitation sessions per day, six days per week. The patients were subjected to rehabilitation interventions using both conventional and robotic approaches, with the objective of enhancing their strength, balance, proprioception, and range of motion (ROM) and of facilitating recovery of ambulation. The type of rehabilitation treatment differed according to the type of surgery received. A team comprising oncologic and plastic surgeons from the Fondazione Policlinico Campus Bio-Medico in Rome and a team of physicians (physiatrists, neurologists), physical therapists, occupational therapists, and biomedical engineers from the Fondazione Don Carlo Gnocchi in Rome developed a customized rehabilitation protocol tailored to the distinct surgical approaches received by the patients (Table 1).

**Figure 1:**
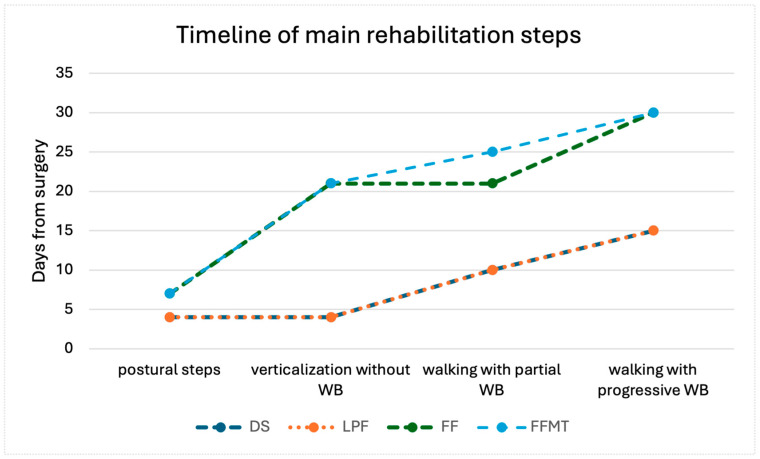
Timeline of main rehabilitation steps. The values displayed in the graph correspond to the mean values of the ranges presented in Table 1. DS: demolition surgery; LPF: local perforator flaps; FF: free flap; FFMT: free functional muscle transfer.

**Figure 2:**
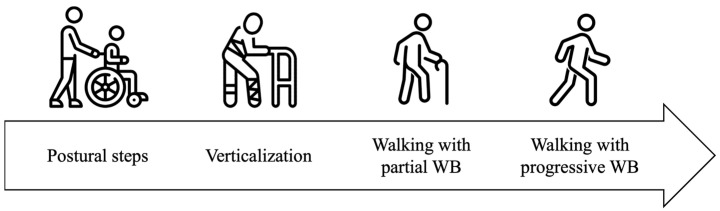
Graphic timeline of main rehabilitation steps. WB: weight bearing.

The protocol was developed on the basis of clinical evidence gained through the experience of the two multidisciplinary teams, as well as by data from the literature. It recommends the timing of the different steps of a rehabilitation program, depending on the type of surgery performed, in accordance with wound healing times, preventing complications from disuse and immobility of the lower extremities. The key rehabilitation steps include postural steps and bed–wheelchair transfers, verticalization, ambulation without weight bearing on the operated limb or with partial bearing, and ambulation with gradual weight bearing with or without a brace. Furthermore, the optimal timing for performing active or passive mobilization exercises of the involved joint with recovery of the range of motion and isometric strengthening of the limbs has been analyzed (Figure 1 and Figure 2).

As illustrated in Figure 1, for patients without reconstruction or with LPF, early rehabilitation starts with postural steps, seated trunk control exercises, and upper limb strengthening within the first week. At this stage of rehabilitation, mobility is restricted to wheelchair use only. Verticalization and passive mobilization of the reconstructed limb can be started between 7 and 15 days from surgery, while walking with partial load is possible after 7–15 days. For patients receiving FF or FFMT, recovery is longer. Verticalization is delayed until after 21 days, and loading activities are introduced cautiously, with a gradual progression beyond 30 days. Rehabilitation of the donor limb, including recovery of range of motion and isometric contractions, begins between 15 and 30 days, while progressive weight bearing on the reconstructed limb may take longer, particularly for FFMT patients, for whom the use of a brace may be necessary.

The rehabilitation protocol was developed considering also different timelines based on the extent of muscle, vascular, tendon, or nerve structures that were involved. In the absence of complications or significant influencing factors, the conventionally accepted healing time for a surgical wound is considered to be between two and three weeks [20]. Springfield posited that the absence of complications in the initial three to four weeks following a surgical wound is associated with a favorable prognosis for complete healing [21]. Consequently, the time required for mobilization of a limb that has not undergone reconstructive surgery is shorter than that of patients who have undergone reconstructions with various types of flaps.

Patients who have undergone surgery in which the lower limb without the sarcoma is not involved (local perforator flap or flaps harvested from other donor sites such as the latissimus dorsi muscle) have faster verticalization and ambulation times due to the ability to distribute the load early on the lower limb without the sarcoma. In the case of FFMT, recovery times are longer overall due to the necessity of preserving the function of the transplanted flap. David Chi and Shreya Raman have also highlighted the need for the limb to be kept immobile for at least four to six weeks, with the use of a brace, in order to ensure the stability of the newly created insertion and origin sites of the transplanted muscle [22].

### 2.4. Clinical Assessment

All patients were clinically evaluated at the time of admission to the ward (T0) and at the discharge (T1) using the modified Barthel Index Scale (mBI), the Numeric Rating Scale (NRS), and the Adapted Patient Evaluation Conference System (APECS):

- The mBI is a 10-item measure of physical disability with a score range of 0 to 100, often used to assess abilities and functioning in activities of daily living [23].
- The NRS, an 11-point (0–10) numerical rating scale, measures the patient’s perceived level of discomfort [24].
- The APECS employs an 8-point rating scale (0–7) to assess walking ability and independence [25].

Of the sample recruited in the study, 18 patients also underwent a 10 Meter Walk Test (10 MWT) once able to walk with or without an aid (T0+) and at discharge (T1). The test assesses walking speed over a short path [26].

### 2.5. Statistical Analysis

Descriptive and inferential analyses were performed using SPSS (version 28, IBM, New York, NY, USA). Due to the limited sample size, non-parametric tests were employed. Specifically, to assess changes in the clinical scales throughout the rehabilitation program, the Wilcoxon signed-rank test was used. For the analysis, a *p*-value less than 0.05 was considered statistically significant.

## 3. Results

Thirty-one patients met the established inclusion criteria and were enrolled in the study. All patients showed compliance and completed the above-reported rehabilitation program. Table 2 provides a summary of the clinical characteristics of the patients and the mean values of the scale at admission, while Table 3 presents the tumor characteristics and the corresponding treatment.

The tumor was localized in the thigh in 80.6% of cases (*n* = 17 antero-medial or antero-lateral and *n* = 8 posterior). Three cases were localized in the retroperitoneum with involvement of the iliopsoas and femoral nerve.

In accordance with the above-mentioned classification, patients underwent different demolitive and reconstructive surgical treatments:

- 15 patients: demolitive surgery without secondary reconstruction (primary closure).
- 3 patients: demolitive and reconstructive surgery with local perforator flaps.
- 5 patients: demolitive and reconstructive surgery with free flaps (harvested from the latissimus dorsi muscle in four cases and the contralateral gracile muscle in one).
- 8 patients: demolitive and reconstructive surgery with FFMT (harvested from the contralateral thigh in four cases, from the latissimus dorsi muscle in three cases, and from the upper limb in one).

Forty-five percent (*n* = 14) of patients underwent radiation therapy, chemotherapy, or hyperthermia. A total of four wound complications were registered during hospitalization (13%). This included one case of wound dehiscence, two cases of delayed wound closure, and one case of occurrence of hematoma.

The average length of stay was 68.5 ± 32.1 days. The average time between surgery and discharge from the rehabilitation ward was 90.7 ± 50.4 days.

**Figure 3:**
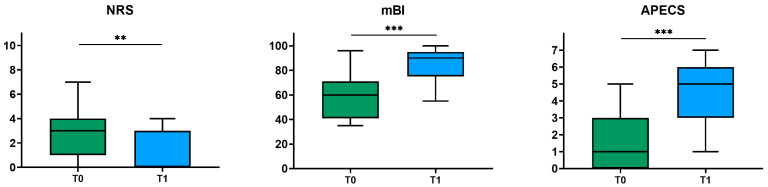
Box plots depicting the modified Barthel Index (mBI), the Adapted Patient Evaluation Conference System (APECS), and the Numeric Rating Scale (NRS) measured at admission (T0) and at discharge (T1). The asterisks refer to the statistical analysis (** *p* < 0.01; *** *p* < 0.001).

**Figure 4:**
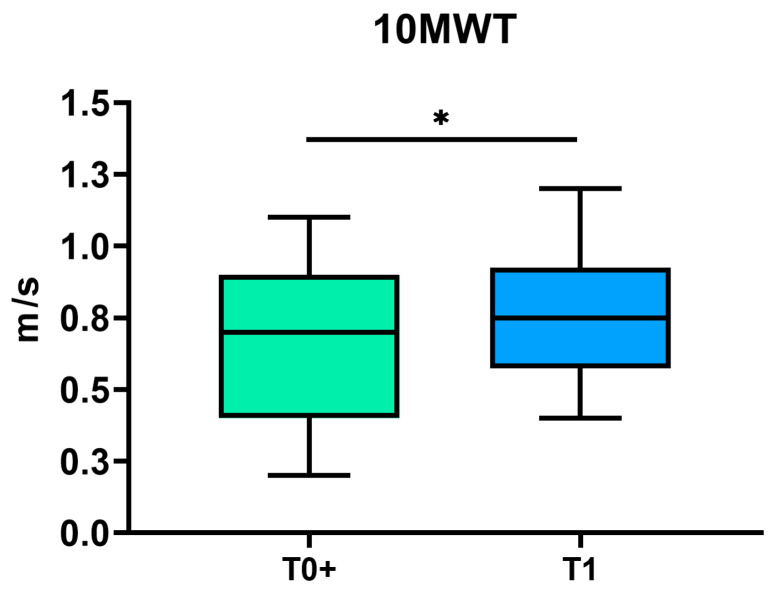
Box plots depicting the 10 Meter Walk Test (10 MWT) measured once patients were able to walk (T0+) and at discharge (T1). The asterisks refer to the statistical analysis (* *p* < 0.05).

The results of the statistical analysis indicated a statistically significant improvement in all the scales analyzed. Specifically, we reported a statistically significant increase in mBI (*p* < 0.001), APECS (*p* < 0.001), and NRS (*p* = 0.01). In the group of patients undergoing the 10 MWT (*n* = 18), a significant increase in walking speed was observed (*p* = 0.012) (Figure 3 and Figure 4).

## 4. Discussion

The aim of this paper was to suggest a surgery-tailored rehabilitation protocol for patients with primary sarcoma of the limbs and to evaluate its effects in a pilot study.

To the best of our knowledge, this is the first study in the literature reporting specific rehabilitation protocols for patients with STSs, particularly differentiated according to the type of demolitive and reconstructive surgery performed. We believe that the type of rehabilitation should be tailored to the specific characteristics of the individual patient, their history, their physical condition prior to surgery, and the goals set. Indeed, the proposed protocol should be utilized as the basis for developing a personalized treatment plan.

As reported by the study by Tobias et al., there is no standard rehabilitation protocol for patients with sarcomas [14]. As proposed in our protocol, the study by Tobias et al. also highlights the critical importance of establishing weight-bearing restrictions in coordination with the surgical team, as well as determining appropriate timing for mobility, postural steps and transfers [14].

Our results showed a significant improvement in all the scales utilized. In particular, the analysis conducted at the conclusion of the rehabilitation program revealed a statistically significant improvement in ambulation (APECS). It has been documented in the literature that the onset of ambulation in patients undergoing surgical procedures of varying types occurs at different times. In the case of patients undergoing microsurgery and functional surgery, for instance, longer recovery periods are necessary to allow for optimal wound healing and tissue rooting [22]. It would undoubtedly be of interest to analyze changes in gait quality following both demolitive and reconstructive surgery by means of gait analysis in future studies.

Additionally, an increase in walking speed was identified in a sub-group of 18 patients who underwent a detailed analysis with the 10 MWT scale. Previously, Atsushi Tanaka examined alterations in walking velocity, as assessed by Maximum Walking Speed (MWS), in a cohort of 15 patients diagnosed with thigh sarcoma [27]. The results demonstrated a significant improvement in speed between three and six months post-surgery, reaching a plateau at 12 months. These results are consistent with our findings of improved walking speed at discharge, which, in the subgroup of our patients, averaged approximately three months after surgery.

The observed improvement in walking provides further justification for the significant increase in the mBI. Indeed, some items on this scale pertain to walking or the ability to move and perform activities of daily living. A review of the literature reveals a limited number of articles that analyze mBI as a functional outcome in oncology patients undergoing rehabilitation. Furthermore, these studies focus on patients undergoing pre-rehabilitation in anticipation of surgery [28]. This scale is often used as a prognostic tool [29,30] or as a measure of functional independence in cancer patients undergoing palliative care [31]. For example, in a retrospective study by Morishima et al. on 12,134 patients with newly diagnosed gastric, colorectal, and lung cancer, the results showed that lower mBI was associated with worse survival, indicating its importance as a prognostic tool [32]. A prospective study of the application of mBI on a larger sample of patients with cancer undergoing rehabilitation for mobility impairment would be a valuable addition to the existing literature.

Subsequently, our study revealed a significant improvement in the pain outcome measure (NRS). Pain in patients with STSs can have various etiologies, including neuropathic pain, which arises from nerve damage during limb salvage, therapy-induced pain, or tumor-related pain. Additionally, patients who have undergone major demolitions, with secondary reconstruction, may experience muscle weakness and alterations in gait analysis and weight-bearing, leading to possible joint pain [16]. The role of a rehabilitation program in the treatment of pain in conjunction with pharmacological therapy in patients with cancer is well documented [33]. It should be noted that our study did not employ a tool capable of differentiating the type of pain experienced by the patient (nociceptive or neuropathic). Further research is required to ascertain whether the type of pain experienced by patients improves following rehabilitative treatment.

It would be interesting to evaluate in the future the maintenance or a further improvement of the outcomes achieved in our patients through rehabilitation, with a long-term follow-up. In this regard, Gerrand et al. and Davis A. highlighted how physical functioning could improve during treatment for about a year before stabilizing [9,34], even if remaining less active than controls [9]. Nevertheless, in our opinion, the high disease recurrence and mortality rates in some histotypes must be considered when evaluating the long-term outcomes of these patients.

In the context of wound healing for patients undergoing surgery, it is essential to balance the time required for wound healing with the need for early mobilization to prevent the occurrence of complications related to immobilization (reduction in range of motion of the joint involved, reduction in the muscle tone and muscle strength).

In our sample, an analysis of the set of surgeries revealed the presence of only one case of delayed wound healing, two cases of other complications of incisional closure, and one case of hematoma. No episodes of skin or fat necrosis or graft rejection were recorded during the period examined.

In a study that analyzed only patients undergoing vascularized flap reconstruction, Spirer et al. also found very low complication rates (4%) [35]. In contrast, O’Sullivan and colleagues observed that the incidence of complications following direct closure was higher than that observed following flap reconstruction surgery (15% vs. 2%) [36], similar to recent findings by Madeleine N. Hoang et al. [37]. Due to the limited size of the pilot study sample, it was not feasible to conduct a comparative analysis of the different surgical procedures.

It is noteworthy that patients undergoing radiation therapy frequently have undergone reconstructive surgery with FF or FFMT. Indeed, Brunetti et al. highlight the importance of microsurgical reconstruction in cases of extensive defects and/or those that have undergone prior radiation therapy [8].

The proposed rehabilitation protocol for patients with STSs has the potential to serve as a valuable tool for the multidisciplinary team (comprising surgeons, oncologists, and rehabilitators) insofar as it provides indications and recommendations for post-operative management. This could help to align expectations and actions, ensuring that each specialist is aware of the patient’s capabilities and limitations at different stages of recovery, thereby preventing complications and improving the quality of rehabilitation.

## 5. Limits

The main limitations of this study are the heterogeneity of the tumor sites and characteristics. Due to its rarity, a significant challenge was presented by the fact that the sample available did not allow for a division and comparison between the various subgroups based on the type of surgery performed and the tumor’s location. Furthermore, another important limitation is the absence of a control group that did not follow the proposed protocol. We acknowledge that the improvements observed at the end of the rehabilitation program may not be directly attributable to the protocol itself and could be influenced by other factors. However, we hope to conduct a comparative study on a larger sample in the future to assess this protocol against other treatment approaches. In addition, the potential impact of comorbidities on the wound healing process and subsequent rehabilitation has to be considered in a larger sample. The suggested rehabilitation protocol could be modified by the presence of comorbidities on the wound healing process. Consequently, this protocol should be regarded as a framework for the development of treatment plans that are tailored to the specific characteristics of individual patients.

## 6. Conclusions

Patients with lower extremity impairments following surgery for STSs who underwent rehabilitation following the proposed protocol, tailored to the type of surgery performed, showed significant improvements in motor performance, daily activity ability, walking, and pain management.

To the best of our knowledge, this is the first rehabilitation protocol suggested for patients with STSs, focusing on walking recovery and ability in activities of daily living, based on demolitive and reconstructive surgery.

## Figures and Tables

**Table 1:** Timelines and modality for postural steps, verticalization, walking, and therapeutic exercises. Days referred to the latency from the surgery.

Rehabilitation Program	Demolition Surgery Without Reconstruction	Local Perforator Flaps	Free Flap *	Free Function Muscle Transfer *
Postural steps and transfers (without weight on the reconstructed limb) Sit trunk control exercises Joint mobility and muscle strengthening of the UL Mobility allowed only by wheelchair	0–7 days	0–7 days	0–15 days	0–15 days
Verticalization (without WB on the reconstructed limb)	0–7 days	0–7 days	>21 days	>21 days
Range of motion gradual recovery of the donor limb	NA	NA	15–21 days	15–30 days
Isometric contractions of the donor limb	NA	NA	>15 days	>15 days
Walking with partial WB on the reconstructed limb	7–15 days	7–15 days	>21 days	20–30 days
Passive mobilization of the reconstructed limb	7–15 days	7–15 days	>15 days	>30 days
Isometric contractions of the reconstructed limb	7–15 days	7–15 days	>15 days	45–60 days
Walking with progressive WB on the reconstructed limb	>15 days	>15 days	>30 days	>30 days (with brace)
Range of motion gradual recovery of the reconstructed limb	>30 days	>30 days	>30 days	>60 days

* Caution needed in mobilization and muscle strengthening exercises if the donor site is different from the lower limb, in accordance with the wound healing time. UL: upper limb; WB: weight bearing.

**Table 2:** Clinical characteristics of the patients and the mean values of the scale at admission.

	Subjects *n* = 31	
Time from the surgery to the admission to the rehabilitation ward (days, Mean ± SD)	Patient without surgical complication (*n* = 25)	7.76 ± 5.0
	Patient with surgical complication (*n* = 6)	82.5 ± 38.3
Length of stay (days, Mean ± SD)	68.5 ± 32.6	
modified Barthel Index (Mean ± SD)	59.6 ± 17.3	
Adapted Patient Evaluation System (Mean ± SD)	1.5 ± 1.5	
Numeric Rating Scale (Mean ± SD)	2.7 ± 2.1	

**Table 3:** Tumor characteristics, surgical procedures, and medical treatment for each patient.

ID	Sex	Age	Tumor	Side	Type of Surgery	Resection	Reconstruction	Medical Therapy	Modified Barthel Index (T0)
1	F	73	Pleomorphic sarcoma	L	FFMT	SM, ST, gracilis	Latissimus dorsi	RT + CT + Hyperthermia	93
2	F	80	Liposarcoma	L	DS	LHBF, adductor brevis	No	No	77
3	M	48	Liposarcoma	R	FF	TFL, sartorius, VM	Latissimus dorsi	CT	63
4	F	73	Atypical lipomatous tumor	R	DS	VM	No	No	56
5	F	53	Leiomyosarcoma	R	FF	VM, VI, RF	Gracilis (controlateral)	CT	85
6	M	77	Liposarcoma	R	DS	Gracilis, adductor longus and brevis	No	RT + Hyperthermia	73
7	M	46	Myxofibrosarcoma	L	FFMT	VM, VI, RF	RF (controlateral)	RT + Hyperthermia	40
8	F	46	Pleomorphic sarcoma	L	DS	VM	No	No	81
9	F	59	Atypical lipomatous tumor	L	DS	VM	No	No	66
10	M	77	Myxofibrosarcoma	L	FFMT	VM, VI, RF	RF (controlateral)	RT + CT + Hyperthermia	36
11	M	59	Pleomorphic sarcoma	L	FFMT	LHBF, VL	Latissimus dorsi	RT + Hyperthermia	38
12	F	79	Malignant peripheral nerve sheath tumors	R	LPF	VM, VI, sartorius, adductor magnus	SCIP	No	43
13	F	50	Leiomyosarcoma	R	LPF	VM, VI, RF	PAP	No	63
14	M	56	Liposarcoma	L	DS	Iliopsoas	No	CT	55
15	M	76	Atypical lipomatous tumor	R	DS	Biceps femoris	No	No	60
16	F	76	Atypical lipomatous tumor	L	DS	VM, VI, VL, RF	No	No	40
17	F	70	Synovial sarcoma	R	FFMT	Tibialis posterior, extensor digitorum longus	Radial flexor of the carpus, palmaris longus	RT+ Hyperthermia	82
18	F	53	Mesenchymal tissue neoplasms	L	LPF	RF, VI	Gracilis	No	96
19	M	32	Synovial sarcoma	R	FFMT	VM, VI, VL	Latissimus dorsi	CT	63
20	M	72	Pleomorphic sarcoma	R	FFMT	VM, VI, VL, RF	RF, VM (controlateral)	RT + CT	71
21	F	53	Synovial sarcoma	L	FF	Plantar muscles	Latissimus dorsi	RT + CT	52
22	M	70	Liposarcoma	R	FF	Sartorius, gracilis, adductor magnus, SM, ST	Latissimus dorsi	RT + CT	40
23	F	72	Dedifferentiated sarcoma	L	DS	Iliopsoas, sartorius, RF, VL, VI	No	No	41
24	M	72	Dedifferentiated sarcoma	L	DS	LHBF, SM	No	No	47
25	F	86	Liposarcoma	L	FF	Extensor hallucis longus, tibialis anterior, soleus	Latissimus dorsi	RT	57
26	F	60	Pleomorphic sarcoma	L	FFMT	RF	VL (controlateral)	RT + Hyperthermia	35
27	F	46	Liposarcoma	L	DS	Iliopsoas	No	No	52
28	M	69	Myxofibrosarcoma	L	DS	VL, TFL	No	No	64
29	M	64	Leiomyosarcoma	R	DS	Iliopsoas	No	No	70
30	F	45	Spindle cell sarcoma	L	DS	SM, ST, LHBF	No	No	70
31	M	72	Atypical lipomatous tumor	L	DS	VM, RF	No	No	40

F: female; M: male; L: left; R: right; FFMT: free functional muscle transfer; DS: demolition surgery; FF: free flap; LPF: local perforator flap; SM: semimembranosus; ST: semitendinosus; LHBF: long head of biceps femoris; TFL: tensor fascia lata; VM: vastus medialis; VI: vastus intermedius; RF: rectus femoris; VL: vastus lateralis; SCIP: superficial circumflex iliac artery perforator; PAP: profunda artery perforator; RT: radiotherapy; CT: chemotherapy.

## Data Availability

The data that support the findings of this study are available from the corresponding author upon reasonable request.
